# The cold pressor test in interictal migraine patients – different parasympathetic pupillary response indicates dysbalance of the cranial autonomic nervous system

**DOI:** 10.1186/s12883-018-1043-2

**Published:** 2018-04-16

**Authors:** Ozan E. Eren, Ruth Ruscheweyh, Christoph Schankin, Florian Schöberl, Andreas Straube

**Affiliations:** 1 Department of Neurology, University Hospital, LMU Munich, Campus Großhadern, Marchioninistr. 15, 81377 Munich, Germany; 2 Department of Neurology, Inselspital, Bern University Hospital, University of Bern, Bern, Switzerland

**Keywords:** Migraine, Pupillometry, Autonomic nervous system (ANS), Parasympathetic nervous system (PSNS), Sympathetic nervous system (SNS), Cold pressor test (CPT)

## Abstract

**Background:**

Data on autonomic nervous system (ANS) activations in migraine patients are quite controversial, with previous studies reporting over- and underactivation of the sympathetic as well as parasympathetic nervous system. In the present study, we explicitly aimed to assess the cranial ANS in migraine patients compared to healthy controls by applying the cold pressor test to a cohort of migraine patients in the interictal phase and measuring the pupillary response.

**Methods:**

In this prospective observational study, a strong sympathetic stimulus was applied to 20 patients with episodic migraine in the interictal phase and 20 matched controls without migraine, whereby each participant dipped the left hand into ice-cold (4 °C) water for a maximum of 5 min (cold pressor test). At baseline, 2, and 5 min during the cold pressor test, infrared monocular pupillometry was applied to quantify pupil diameter and light reflex parameters. Simultaneously, heart rate and blood pressure were measured by the external brachial RR-method at distinct time intervals to look for at least clinically relevant changes of the cardiovascular ANS.

**Results:**

There were no significant differences between the migraine patients and controls at baseline and after 2 min of sympathetic stimulation in all the measured pupillary and cardio-vascular parameters. However, at 5 min, pupillary light reflex (PLR) constriction velocity was significantly higher in migraineurs than in controls (5.59 ± 0.73 mm/s vs. 5.16 ± 0.53 mm/s; unpaired t-test *p* < 0.05), while both cardiovascular parameters and PLR dilatation velocity were similar in both groups at this time point.

**Conclusions:**

Our findings of an increased PLR constriction velocity after sustained sympathetic stimulation in interictal migraine patients suggest an exaggerated parasympathetic response of the cranial ANS. This indicates that brainstem parasympathetic dysregulation might play a significant role in migraine pathophysiology. More dedicated examination of the ANS in migraine patients might be of value for a deeper understanding of its pathophysiology.

**Electronic supplementary material:**

The online version of this article (10.1186/s12883-018-1043-2) contains supplementary material, which is available to authorized users.

## Background

A hallmark of migraine attacks is a concomitant variety of vegetative symptoms, such as loss of appetite, nausea or vomiting, with some patients even showing signs of activation of the cranial autonomic nervous system (e.g. parasympathetic system), such as lacrimation, sweating, rhinorrhea or nasal congestion [1–3]. Furthermore, there is increasing evidence that modulation of the parasympathetic nervous system might be useful in the prevention of, or the cessation of migraine attacks [4, 5]. Otherwise, previous studies of autonomic function in migraine showed inconclusive and even conflicting results regarding the role and interaction of the sympathetic and parasympathetic system.

The advantages of pupillometric testing are that it a) assesses both the sympathetic and parasympathetic innervation simultaneously concerning the pupillary reflex, and that it is b) a well-established method to evaluate autonomic function in the innervation area of the cranial nerves for various conditions [6–8]. The analyses of heart rate and blood pressure allow for at least a rough and clinically relevant evaluation of the cardiovascular autonomic nervous system (ANS). In the present study, we directly tested cranial and cardiovascular autonomic responses of migraineurs in the interictal phase during sustained sympathetic stimulation by the cold pressor test (Additional file 1). We thereby specifically tested the hypothesis whether migraine patients, when compared to age- and gender-matched controls, show different autonomic responses of the cranial ANS in the interictal phase as measured by pupillary response.

## Methods

The experiments were performed at the Department of Neurology, University Hospital Munich, Campus Großhadern. The study was conducted in accordance with the Declaration of Helsinki and approved by the ethics committee of the medical faculty of the Ludwig-Maximilians-University Munich (*No. 133–13)* and all patients gave their written informed consent.

### Subjects

All subjects were interviewed by a headache specialist and had a thorough, standardized neurological examination by a senior neurologist. All subjects had an unremarkable medical history except for headache in the migraine group.

Inclusion criterion was a diagnosis of episodic migraine with or without aura in accordance with the “International Classification of Headache Disorders 3 Beta” (IHCD-III beta) [1]. The migraine group consisted of 20 patients (14 women, age range 23 to 33 years, mean age 26.9 ± 2.5 years) who were tested interictally, i.e. during non-headache periods with at least 48 h without headache before and after testing. All the 20 included migraine patients had a low to moderate attack frequency (in total 10.3 ± 10.4 in the last three months). 19 of the 20 migraineurs did not have prophylactic treatment ever; and one patient stopped his medical prophylaxis more than three months ago. Furthermore, all 20 patients did not take any acute medication in the last 14 days before autonomic testing within the study. Exclusion criteria were a past history of autonomic dysfunction such as syncope or postural orthostatic tachycardia syndrome (POTS), a history of corneal or conjunctival disease, as well as anisocoria (difference of the pupil diameter larger than 1 mm), or any other pathological findings in the neurological examination [9]. Furthermore, the subjects were tested interictally, i.e. during non-headache periods with at least 48 h without headache before and after testing.

The control group consisted of 20 subjects (10 women, age range 23 to 34 years, mean age 28.7 ± 3.5 years). No one of the healthy controls fulfilled the criteria of migraine [1] or any other primary headache. Since you only rarely find subjects, who are completely free of any headaches, at least some in the control group had a history of occasional headaches such as headache related with a flu or after alcohol exposure etc.

### Psychometric measurement: Migraine Disability Assessment (MIDAS)

The MIDAS was filled in by all subjects (also the control group) and is a well-known and often used self-administered patient questionnaire to assess the impact of headache on daily life [10]. There are three scores generated: MIDAS total (impact of headache on the abilities of everyday life), MIDAS A (number of headache days in the last 3 months) and MIDAS B (average pain intensity 0–10).

## Experimental measurements

### Cold pressor test (CPT) / cardiovascular parameters

In general, the cold pressor test is used in clinical routine to assess the function of the ANS and the left ventricle. Temperature is a known stressor to affect heart rate (HR) and blood pressure (BP), therefore painful cold stress leads to a spike in activation of the sympathetic nervous system and consequently to the release of norepinephrine. The resulting pressor response is defined by an increase in HR and BP [11]. There is no standardized scheme to perform the cold pressor test. Traditionally, it is performed by dipping the left hand into 0–4 °C iced water (to the wrist, fingers spread) for 1–5 min. To increase sensitivity, we decided to extend the observation period up to 5 min and used 4° iced water [11–15].

To roughly assess the response of the cardiovascular autonomic nervous system we measured the HR and the BP with the conventional external brachial RR-method on the right arm at fixed time points.

### Pupillometry

Pupillary function was assessed with the monocular infrared “Compact Integrated Pupillograph (CIP)” (AMTech Pupilknowlogy GmbH, Heidelberg, Germany). Subjects were seated comfortably in front of the CIP. The pupil was automatically detected by the infrared camera and the diameter was measured continuously over 2 s at a sampling frequency of 250 Hz. The light stimulus to measure the PLR was conducted by the integrated LED with an intensity of 10,000 cd m^− 2^ and a duration of 200 ms [16] and was repeated 5 times with an interval of 10 s. Measurements started after 5 min of light adaptation and subsequent 10 min of dark adaptation. The following parameters were registered automatically for the left eye:

1. **Pupillary constriction velocity in mm s**^**− 1**^.
2. **Pupillary dilatation velocity (slow) in mm s**^**− 1**^**,** i.e. velocity of pupillary dilatation at the end of the dilatation phase.
3. Latency time in ms, i.e. the period from the initiation of the light stimulus until the start of pupillary constriction.
4. Pupillary dilatation velocity (fast) in mm s^− 1^, i.e. velocity of pupillary dilatation at the beginning of the dilatation phase.
5. Amplitude in mm, i.e. maximum change of pupillary diameter.
6. Initial diameter in mm, i.e. pupillary diameter at the beginning of the measurement.

In order to assess the response of the ANS during the cold pressor test, we decided to analyze – in accordance with the existing literature [6–9, 16] – the pupillary constriction velocity for the parasympathetic response and slow pupillary dilatation velocity for the sympathetic response. In addition, we obtained the mean pupillary diameter for both eyes at baseline and after the cold pressor test.

### Pain assessment

We obtained standardized pain ratings with a numerical rating scale (NRS), from 0 (i.e. no pain) to 10 (i.e. maximum pain), of 20 subjects (10 in each group) to evaluate whether any measured differences in the ANS are a consequence of differences in pain perception.

### Statistical analysis

Data at three distinct time points were registered: T0: baseline before the cold pressor test; T1: two minutes during the cold pressor test; T2: five minutes during the cold pressor-test; T1’ as additional time point only for the measurement of blood pressure; i.e. immediately after dipping the hand into the iced water. All the obtained data is given as mean ± SD. For the pupillary response, each data point was an average of at least five subsequent registrations of the pupillary light reflex (one every 10 s). For the cardiovascular parameters, mean blood pressure and heart rate were obtained once at each time point.

Statistical analysis was done using SPSS 22.1 (IBM Corp. Released 2013. IBM SPSS Statistics for Windows, Version 22.1. Armonk, NY: IBM Corp.). The data distribution was parametric as tested by the Kolmogorov-Smirnov (K-S) test. Repeated analysis of variance (ANOVA) was used to compare parameters between multiple time points within both groups (migraine, control). We performed posthoc Bonferroni-correction for multiple testing. Whenever we found a significant effect in the ANOVA, then we applied an unpaired t-Test at any timepoint(s) (T0, T1, T2) to detect the exact time point(s) (T0, T1, T2) of the statistical difference. Statistical significance was considered at *p* < 0.05.

## Results

As expected, the MIDAS scores were significantly higher in the migraine group (t-test, *p* < 0.01) with a significant impact of headache on the abilities of everyday life (MIDAS total: 12.25 ± 21.39 vs. 0.40 ± 1.10), a significantly higher number of headache days in the last 3 months (MIDAS A: 10.37 ± 10.93 vs. 1.30 ± 1.49), as well as an increased pain score (MIDAS B: 6.00 ± 2.11 vs. 1.75 ± 1.80) in the migraine group. A total MIDAS score above 10 indicates moderate disability.

There were no adverse effects in the control group. In the migraine group one female suffered from a syncope shortly after T2. The data are summarized in Table 1.

**Table 1:** Patient characteristics

Group	Migraine (*n* = 20)	Controls (*n* = 20)	*p*-value
Age	26.90 ± 2.45	28.7 ± 3.50	n.s.
Sex	14 female / 6 male	10 female / 10 male	n.s.
MIDAS A	10.37 ± 10.93	1.30 ± 1.49	*p* < 0.05
MIDAS B	6.00 ± 2.11	1.75 ± 1.80	*p* < 0.05
MIDAS total	12.25 ± 21.39	0.40 ± 1.10	*p* < 0.05

### Pupillary response

The pupil diameters at baseline were not different between both groups (migraineurs: 7.22 ± 0.64 mm vs. controls: 7.18 ± 0.8 mm).

**Fig. 1:**
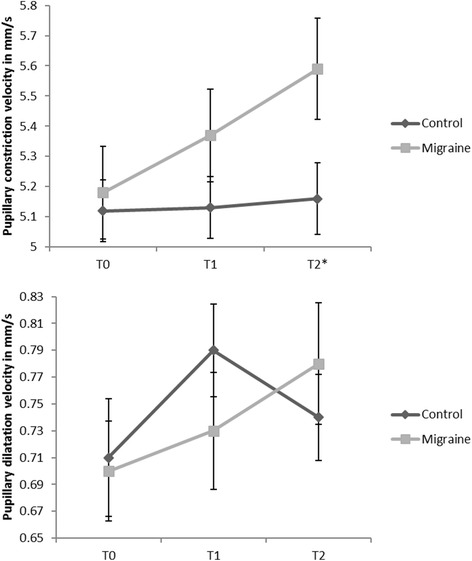
Pupillary response. T0: baseline, T1: 2 min during cold pressor test, T2: 5 min during cold pressor test. Additional for cardiovascular response: T1’: immediately after starting cold pressor test

Regarding pupillary constriction velocity during the light reflex (mm/s), there was a significant interaction between time and group (F = 4.26, *p* < 0.05) due to a continuous increase in pupillary constriction velocity in migraine patients, but not in controls (Ctr) from T0 to T2: T0 (Ctr: 5.12 ± 0.46 vs. migraineurs: 5.18 ± 0.70 mm s^− 1^), T1 (Ctr: 5.13 ± 0.46 vs. migraineurs: 5.37 ± 0.66 mm s^− 1^) and T2 (Ctr: 5.16 ± 0.53 vs. migraineurs: 5.59 ± 0.73 mm s^− 1^) (Fig. 1).

Posthoc analysis by the Bonferroni-correction revealed that the migraine patients had a significantly faster constriction velocity at T2 (*p* = .003), but not at T0 or T1. We further applied an unpaired t-test to compare both groups at each time point separately. There was a significant effect for T2 (*p* = 0.0.42), while T0 (*p* = 0.82) and T1 (*p* = 0.33) were not different between both groups.

For the slow pupillary dilatation velocity (mm s^− 1^) after the light reflex, there were no significant main effects for time or group and no significant interaction between time and group (F = 1.41; *p* = 0.26) from T0 to T2: T0 (Ctr: 0.72 ± 0.20 vs. migraineurs: 0.70 ± 0.16 mm s^− 1^), T1 (Ctr: 0.78 ± 0.15 vs. migraineurs: 0.73 ± 0.18 mm s^− 1^) and T2 (Ctr: 0.74 ± 0.14 vs. migraineurs: 0.77 ± 0.20 mm s^− 1^) (Fig. 1).

### Cardiovascular response

**Fig. 2:**
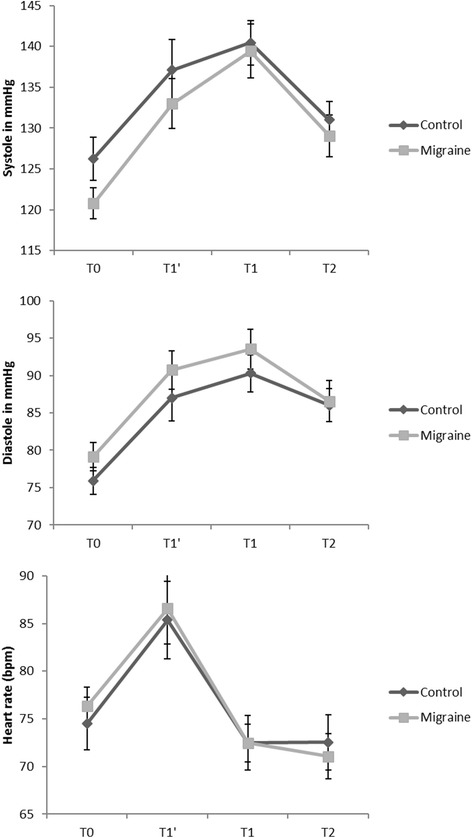
Cardiovascular response. T0: baseline, T1: 2 min during cold pressor test, T2: 5 min during cold pressor test. Additional for cardiovascular response: T1’: immediately after starting cold pressor test

There was a significant effect of time on the cardiovascular autonomic response in all three parameters (*p* < 0.01). However, there were no significant differences between groups and no significant interactions between time and group (systole F = 0.61 *p* = 0.55; diastole F = 1.02 *p* = 0.37 and heart rate F = 0.56 *p* = 0.58) (Fig. 2).

### Pain ratings

**Fig. 3:**
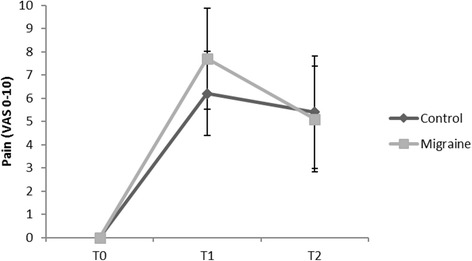
Pain ratings. T0: baseline, T1: 2 min during cold pressor test, T2: 5 min during cold pressor test. Additional for cardiovascular response: T1’: immediately after starting cold pressor test

We obtained ratings of maximal pain with a numerical rating scale (0–10 at T0, T1 and T2) from 20 subjects (10 from each group). There was a significant effect of time on the pain ratings (p < 0.01). But we did not find any significant differences between both groups and no significant interaction between time and group (F = 0.21 *p* = 0.143) (Fig. 3). Further, we did not find any significant correlation between the pain ratings and pupillary constriction velocity at any time point.

## Discussion

Pupillary size and changes in pupillary size depend on many factors (e.g. time, light, environment, sleepiness, emotional state etc.), but reflect in general the balance between the sympathetic (primarily dilatation) and parasympathetic (primarily constriction) nervous system tonus.

At baseline (T0) there was no significant difference in the pupillary and cardiovascular parameters between the migraineurs and the controls, indicating that there are no profound changes in the ANS of migraine patients under normal circumstances. This is in line with a recently published study by Cambron et al., who did not find differences of pupil parameters in migraine patients, neither in the interictal phase nor during migraine attacks [7]. However, at T2 (i.e. five minutes after sympathetic stimulation), the constriction velocity was significantly higher in the migraine patients. This might indicate that the ANS is at least slightly dysregulated in migraine patients also in the interictal, non-headache phase and that sympathetic stimulation can unravel this difference in ANS thresholds. However, the results of previous studies on the ANS thresholds and changes in migraine patients are inconclusive and partially conflictive. At first glance, in great contrast to our data, Mylius et al. showed a significantly slower constriction velocity and a smaller amplitude of pupil constriction within two days after an attack in migraine patients, thus inferring parasympathetic hypofunction [16]. However, for comparison with our data, one has to recognize that the time points of ANS-measurements were different in both studies. While they made their measurements within two days after a migraine attack, this time period was an exclusion criterion for our study, where measurements only more than two days after an attack were recorded. Thus, the data might be conclusive, since migraine patients might suffer parasympathetic dysregulation in the following way: a) lower parasympathetic thresholds under normal circumstances with activation by sympathetic stimulation, as we have shown; b) obvious parasympathetic hyperactivation during migraine attacks possibly triggered by pain in the attack, or vice versa, as a fundamental condition in the pathophysiology of migraine headache [16]; and c) parasympathetic hypofunction directly postictal after the attack, as shown by Mylius [16]. Moreover, Drummond et al. also argued for an increase of the parasympathetic tone during a migraine attack directly related to trigeminal-parasympathetic reflexes, when observing the dilatation of dermal blood vessels during attacks [17].

Corresponding to our findings, a previous study by Tassorelli et al. (15) demonstrated a miotic phase with a maximum at five minutes during the cold pressor test after an initial very short mydriasis in healthy volunteers. Our dataset implies that this physiological parasympathetic pupillary response to the cold pressor-test is more pronounced in migraine patients. This might indeed be an indirect correlate of at least slight parasympathetic dysregulation in migraine.

Pupil dilatation to baseline directly follows pupil constriction. This redilatation process can be divided into two phases: the initial and rapid redilatation phase is rather an effect of withdrawal of the parasympathetic tone than sympathetic activation, whereas the later and slower dilatation phase seems to be an active process induced by peripheral sympathetic innervation [6]. Altogether, we did not record any significant differences between the migraineurs and Ctr in this two-staged pupil dilatation process. However, analyzing the time course of the dilatation process more precisely, there was a slight delay in reaching the maximum dilatation velocity in the migraine group, while velocity itself was unchanged. The migraine group reached the highest dilatation velocity at T2, whereas the Ctr did so at T1, which may be interpreted in terms of a slight dysbalance towards the parasympathetic nervous system (PSNS) in migraine patients. Taken together our findings and the results of the previous studies, there is sufficient evidence of slight dysregulation of the parasympathetic cranial ANS in migraine patients.

Which pathophysiological mechanisms besides a primary cranial autonomic dysregulation might also contribute to the observed differences in cranial autonomic response between the migraine patients and healthy controls: First, it could be due to a difference of peripheral sensory perception and/or central pain processing. Previous studies could demonstrate cutaneous allodynia (CA) for usually not painful sensory stimuli, particularly thermal stimuli, in more than half of patients with episodic migraine during a migraine episode [18, 19]. One study even could show such changes in migraine patients prior to an episode [20]. It is generally accepted that such cutaneous allodynia is a consequence of central sensitization of pain processing pathways and an impairment of the descending pain inhibitory pathways [18, 21–23]. In fact, these mechanisms can lead to a vicious circle in that sense that recurrent migraine attacks can promote central sensitization, which in turn impairs diffuse noxious inhibitory control (DNIC) [23]. Thus, changes of central sensitization and the descending inhibitory pathways could contribute to the observed differences between migraineurs and the healthy controls by perceiving the cold stimulus during the CPT “more painful”. However, comparable pain rating scores between both groups argue against that hypothesis but cannot definitely exclude it.

Secondly, habituation of sensory stimuli, which is mainly a thalamo-neocortical process, can play also a role. Previous studies have shown that migraineurs have deficits in sensory habituation after repeated stimuli of different sensory modalities (i.e. visual, somatosensory) even in the interictal phase [24, 25]. Coppola et al. [26] were able to show, that CPT can significantly change habituation of visually evoked potentials in healthy controls, but not in migraineurs indicating less plasticity of sensory cortical areas. This could result in a faster habituation of the cold stimulus by the CPT in healthy subjects as compared to the migraineurs thus successfully preventing a further continuous increase of pupillary constriction velocity, as shown by our study. However, one would expect that such a habituation deficit is not that specific affecting only constriction velocity, while dilatation velocity not.

Regarding the higher lifetime rate of syncopes (migraineurs: 46% vs. Ctr: 31%) and particularly a higher lifetime risk for repeated syncopes (migraineurs: 13% vs Ctr: 5%) in migraine patients, changes in the cardiovascular autonomic system should be expected [27]. Since we focused on the cranial ANS of migraine patients in that study, we only performed basic cardiovascular monitoring by measuring blood pressure and heart rate at different time points; however, we did not explicitly apply continuous blood pressure measurements and also did not perform analysis of the heart rate variability. The obtained basic cardiovascular responses (i.e. blood pressure and heart rate) to the cold pressor test were comparable in migraine patients and Ctr. The diastole was slightly (not significantly) increased in migraine patients compared to Ctr. Further subclassifying by headache disability by the MIDAS, we observed an only marginally higher resting state diastolic blood pressure in more disabled migraineurs. Shechter et al. explicitly compared three different groups (i.e. disabling migraine, non-disabling migraine and healthy controls) and also did not find significant differences between the three groups when comparing blood pressure response to a psychological stressor.

Cortelli et al. did not find any impairment of the autonomic control of the cardiovascular system in migraineurs interictally [28]. Domingues et al. used two different protocols to provoke a cardiovascular autonomic response, one by mental stress and one by CPT. The latter one was quite similar to our scheme and they also could not find a difference in heart rate and blood pressure after CPT in migraineurs compared to healthy controls [29]. Daluwatte et al. [30] addressed this question of coupling the cranial with cardiovascular ANS in a cohort of healthy children. They also did not find any correlation between PLR and heart rate variability (HRV), despite significant changes in HRV during the different PLR phases [30].

In our study migraine patients and Ctr did not exhibit any differences in sympathetic or parasympathetic regulation of the cardiovascular response at any timepoint (T0-T2) during the CPT. But as already mentioned, we did not apply the necessary gold standard measurements (heart rate variability and continuous blood pressure measurements) therefore to really make clear statements about that issue.

### Limitations

One major limitation of this study is that as we did not have a continuous registration of blood pressure. Thus, we indeed cannot completely exclude clinically relevant fluctuations of blood pressure. We also did not analyze the power spectrum of heart rate variability by using electrocardiography (ECG), which could give further insight into ANS regulatory processes, as the extern brachial method with punctual measurement is not sensitive enough for this purpose. But we explicitly concentrated on the cranial ANS response during cold pressor-testing and only wanted to exclude major changes within the cardiovascular system such as presyncope/syncope, which in turn might affect the cranial ANS response. Furthermore, regarding the influence of emotions, food intake, and cortisol levels on the ANS, we did not explicitly randomize for these factors. Since it is very difficult to find healthy subjects, which are completely free of headaches, we also included such subjects in the control group with a history of occasional headache not fulfilling the current criteria of migraine or any other primary headache. This is also the reason, why we applied the MIDAS score in the control group and indeed found an increased total score of 0.40 ± 1.10. However, this score was really significantly different to the migraineurs and means at least only mild disability. Furthermore, the score matched with the self-rating reports of the healthy controls as not being a “headache patient”.

## Conclusion

In summary, our data indicate different activation thresholds of the cranial ANS in migraineurs in the interictal phase during sympathetic stimulation. Based on our findings, the role of upper brainstem parasympathetic dysregulation in the pathophysiology of migraine should be further examined and elucidated in more detail. Particularly, the underlying differences in somatosensory processing on the level of cerebral networks and neuronal ensembles might be a target for future therapy strategies.

## Key findings

- There is a difference in the autonomic control of the pupillary light reflex in migraine patients outside an attack compared to healthy controls during a sympathetic stimulus.
- There is probably no such difference in the autonomic cardiovascular response, indicating anisotropy of the cranial and cardiovascular ANS in migraine patients.
- There seems to be a more selective role of parasympathetic dysregulation in the cranial innervation area in the pathophysiology of migraine.

## Additional file

Additional file 1: The cold pressor test in interictal migraine patients. It provides the anonymized data of pupillary, cardiovascular and pain response. (XLSX 17 kb)

## Abbreviations

ANS: Autonomic nervous system
BP: Blood pressure
CA: Cutaneous allodynia
CIP: Compact Integrated Pupillograph
CPT: Cold Pressor Test
Ctr.: Controls
ECG: Electrocardiography
HR: Heart rate
IHCD-III beta: International Classification of Headache Disorders 3 Beta
MIDAS: Migraine Disability Assessment
NRS: Numerical rating scale
PLR: Pupillary light reflex
POTS: Postural orthostatic tachycardia syndrome
PSNS: Parasympathetic nervous system
SNS: Sympathetic nervous system
TRPM8: Transient receptor-potential M8
